# EPILAT-IRA Study: A contribution to the understanding of the epidemiology of acute kidney injury in Latin America

**DOI:** 10.1371/journal.pone.0224655

**Published:** 2019-11-14

**Authors:** Raúl Lombardi, Alejandro Ferreiro, Rolando Claure-Del Granado, Emmanuel A. Burdmann, Guillermo Rosa-Diez, Luis Yu, Mauricio Younes-Ibrahim, Cristina Carlino, Jonathan S. Chávez-Iñiguez, Mariana B. Pereira, Carlos F. Varela, Welder Zamoner, Diego Janiques, Soledad Lecueder, Víctor Cerrón-Millán, Alfonso Cueto-Manzano, for the EPILAT-ITA Study Group

**Affiliations:** 1 Dept. of Nephrology, School of Medicine, Universidad de la República, Montevideo, Uruguay; 2 Division of Nephrology, Hospital Obrero #2 - C.N.S., Universidad Mayor de San Simon School of Medicine, Cochabamba, Bolivia; 3 LIM 12, Division of Nephrology, University of Sao Paulo Medical School, Sao Paulo, Brazil; 4 Dept. of Nehrology, Hospital Italiano, Buenos Aires, Argentina; 5 Dept. of Internal Medicine, University of Rio de Janeiro, Rio de Janeiro, Brazil; 6 Hospital Provincial, Rosario, Santa Fe, Argentina; 7 Hospital Civil de Guadalajara, Centro Universitario de Ciencias de la Salud, Jalisco, Mexico; 8 Nephrology Division, Hospital do Servidor Público Estadual, Universidade Nove de Julho, Sao Paulo, Brazil; 9 Botucatu School of Medicine, São Paulo State University-UNESP, Sao Paulo, Brazil; 10 Clinica Renalle, Pontifícia Universidade Católica, Petrópolis, Rio de Janeiro, Brazil; 11 Sanatorio Casa de Galicia, Montevideo, Uruguay; 12 Hospital Nacional Daniel Alcides Carrión, Dept. de Medicina, Servicio de Nefrología, Callao- Lima, Perú; 13 Medical Research Unit of Renal Diseases, Hospital de Especialidades, CMNO, IMSS, Guadalajara, Mexico; Azienda Ospedaliero Universitaria Careggi, ITALY

## Abstract

**Introduction:**

Epidemiology of acute kidney injury (AKI) is highly dependent on patient characteristics, context and geography. Considering the limited information in Latin America and the Caribbean, we performed a study with the aim to contribute to improve its better understanding.

**Methods:**

Observational, prospective, longitudinal, multinational cohort study addressed to determine risk factors, clinical profile, process of care and outcomes of AKI in the region. Patients meeting KDIGO AKI definition were included over a 9-month period and designated community or hospital-acquired. De-identified clinical and lab data were entered in a specifically designed on-line platform. Co-variables potentially linked to AKI onset, in-hospital and 90-days mortality, were recorded and correlated using a multiple logistic regression model.

**Results:**

Fifty-seven physicians from 15 countries provided data on 905 patients, most with acceptable basic needs coverage. Median age 64 (50–74) yrs; most of them were male (61%) and mestizos (42%). Comorbidities were present in 77%. AKI was community-acquired in 62%. Dehydration, shock and nephrotoxic drugs were the commonest causes. During their process of care, 77% of patients were assessed by nephrologists. Kidney replacement therapy (KRT) was performed in 29% of cases. In-hospital mortality was 26.5% and independently associated to older age, chronic liver disease, hypotension, shock, cardiac disturbances, hospital-acquired sepsis, KRT and mechanical ventilation. At 90-days follow up partial or complete renal recovery was 81% and mortality 24%.

**Conclusions:**

AKI was mainly community-acquired, in patients with comorbidities and linked to fluid loss and nephrotoxic drugs. Mortality was high and long-term follow up poor. Notwithstanding, the study shows partially the situation in the participant countries rather than the actual epidemiology of AKI in Latin America and Caribbean, a pending and needed task.

## Introduction

Acute kidney injury (AKI) is a serious public health issue, which changed from the classical paradigm of a hospital-associated condition managed mainly by nephrologists and intensivists, to a broader and multidimensional condition in the context of an integrated health delivery system [1,2]. Recognition of risk factors and causes of AKI is essential in the fight against this syndrome, whose varied epidemiological profile is highly dependent on patient characteristics and the setting in which occurs.

Available information on epidemiology and risk factors for AKI in Latin America is scarce. More studies designed to achieve a better understanding of the magnitude, specific risk factors, regional causes, clinical features, process of care, and outcomes of AKI are required [3,4]

Therefore, the AKI Committee of the Latin American Society of Nephrology and Hypertension (SLANH in Spanish) carried out an epidemiological study in Latin America and the Caribbean in order to assess the profile of the AKI according to the geographical distribution and its relationship with social, economic, sanitary, and cultural conditions.

## Methods

This is an observational, prospective, longitudinal, multinational cohort study performed from November 1^st^ 2017 to July 31 2018. Invitation to participate in the study was made through the webpage of SLANH, personal email sent to members of *RedIRA* (Latin American AKI-Network) and call through local Societies of Nephrology. Participation in the study was voluntary, without any incentive or economic benefit for patients or investigators.

Inclusion criteria: all adult patients and children with confirmed AKI of any etiology diagnosed during the recruitment period.

Exclusion criteria: Patients with chronic kidney disease (CKD) stage 5, patients on chronic dialysis or living with functioning transplanted kidney.

Bioethical considerations: the study protocol was approved by the Ethics Committee of the Hospital de Clínicas, School of Medicine, Universidad de la República, Uruguay (contact Dr. Leticia Cuñetti, lcunetti@gmail.com), and all participants obtained the approval of a local Ethics Committee. The informed consent was considered not mandatory by the reference Ethics Committee given the observational characteristic of the study. However, an informed consent form was available if required by the local Ethics Committee. Study protocol and forms are available on the study’s website (http://estudioira.slanh.net). Data were entered online by the participants in a platform specifically designed for this purpose. Confidentiality of information was appropriately protected by de-identification of data. Once the patient was included in the platform by the participant (the only one aware of patient’s identity), the system assigned randomly a number that identified hereafter the case. Participants had access to the information of their own patients solely through that number.

### Definitions

Modified KDIGO creatinine-based AKI criteria [5] were used for the diagnosis and staging of AKI, specifically increase or decrease in serum creatinine (SCr) ≥ 0.3 mg/dl within 48 hours, or increase or decrease in SCr ≥ 50% with respect to the first available SCr (reference SCr) or the known baseline value within the seven prior days. Renal recovery was considered complete when SCr returned to baseline or reference value or lower, and partial recovery when SCr decline was ≥0.3 mg/dL but did not reach the baseline or reference SCr. Non-recovery was established when SCr did not decrease or the patient remained on dialysis. AKI was considered as community-acquired (CA-AKI) when patient had an elevated SCr within the first 24 hours of admission and hospital-acquired (HA-AKI) when AKI developed during hospital stay.

Acute kidney disease as cause of AKI include glomerular disease, vascular disease, interstitial disease and intrarenal obstruction.

### Collected variables

Data were obtained from the clinical record of patients. Variables included country and city of residence, demographics, socioeconomic condition (housing, availability of drinking water, sanitation, and electricity), accessibility to sanitary system, risk factors and etiology of AKI, hospitalization setting, process of care, kidney replacement therapy, in-hospital complications, discharge condition, and mortality and renal function at 90 days after discharge at outpatient consultation. Serum Cr was requested at five points of the disease course: 1) initial reference value, corresponding to the first available data; 2) level at the time of diagnosis of AKI; 3) maximum level (peak) observed during hospitalization; 4) level prior to the first dialysis in treated patients; and 5) level at discharge in survivors.

### Statistical analysis

Dimensional variables are presented as mean ± standard deviation (SD) or median and interquartile range (IQR) according to their distribution, and the categorical variables as number and proportions. Kolmogorov-Smirnov test was used to explore the data distribution. For the bivariate comparisons between groups, the Chi square test was used for categorical variables and the Student *t* test for quantitative variables. Odds ratio and 95% Confidence Interval (CI) were also calculated. Statistical tests were two-sided and significance was considered with a probability of null hypothesis ≤5%. A multiple logistic regression model was performed to predict mortality using the forward conditional elimination method. The probability for entering the model was set at 0.10 and for elimination at 0.20. Variables that reached a significance <0.10 in the univariate analysis were included. The same procedure was used to characterize the profile of patients with and without consultation with nephrologist. The statistical package IBM SPSS Statistics Base version 22 NY, USA, was used for data processing and for statistical analysis.

## Results

### 1. Study participants

**Fig 1:**
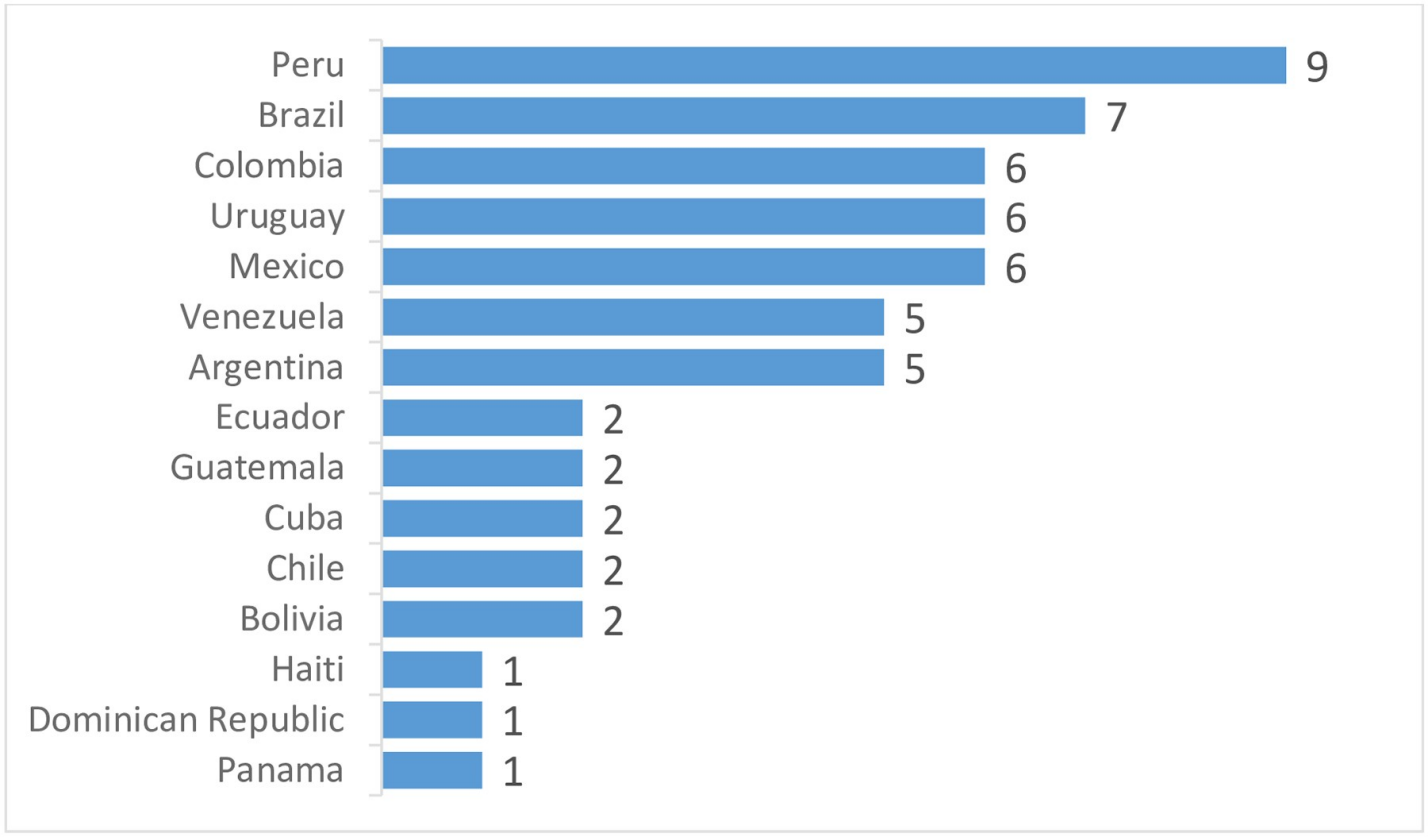
Participants per country. The number of participants per country was heterogeneous, with Peru and Brazil being the most relevant.

Fifty-seven physicians coming from 15 countries involving 41 cities participated in the study (Fig 1. **Participants per country**). Thirty-four (60%) were working in public institutions at the time of the study, 11 (19%) in private institutions and 11 (19%) in university hospitals. Forty-four out of 57 participants (77%) were nephrologists.

### 2. Patients

#### Demographic characteristics

**Fig 2:**
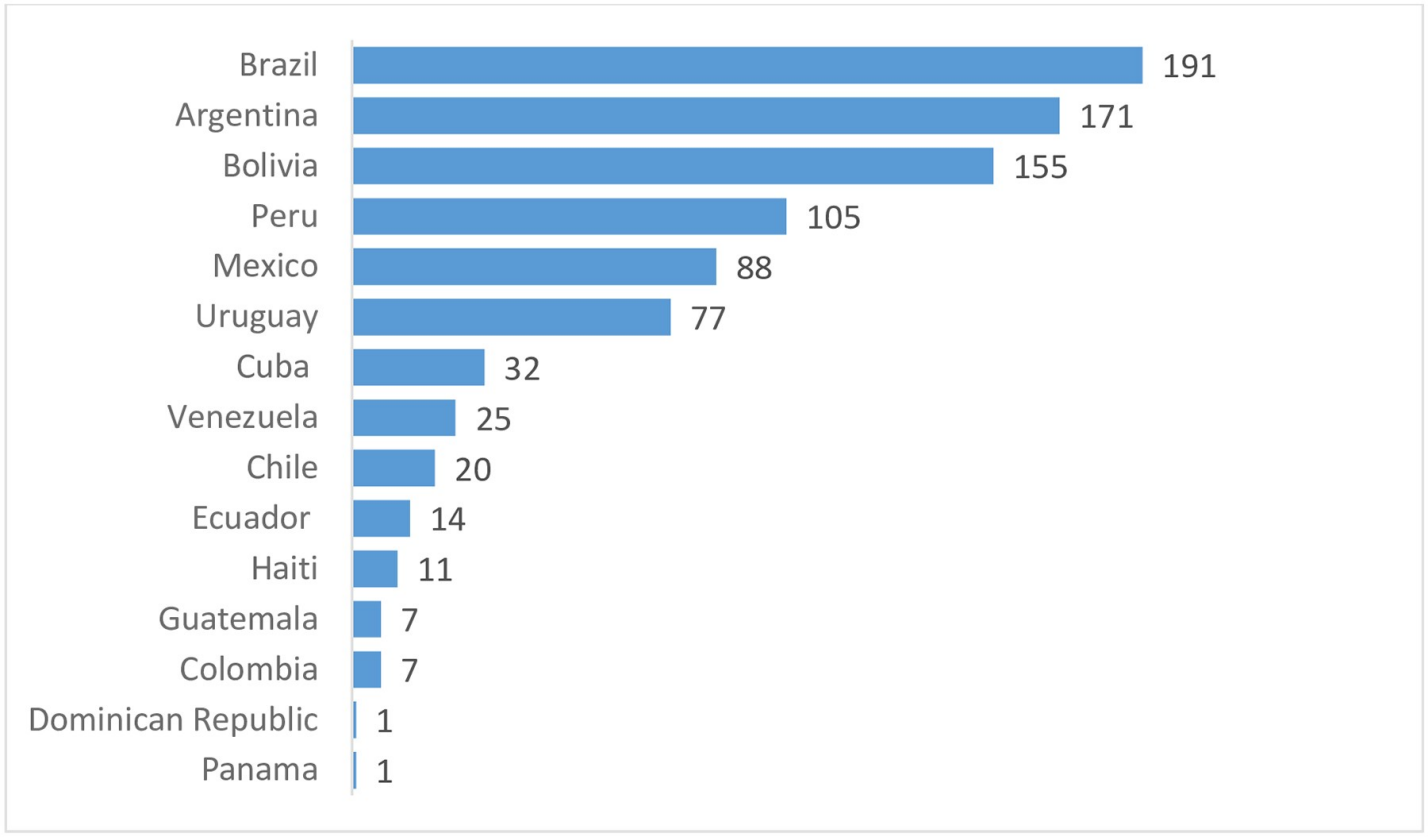
Number of patients per country. The distribution of patients by country was also heterogeneous but presented a different profile than the participants.

**Fig 3:**
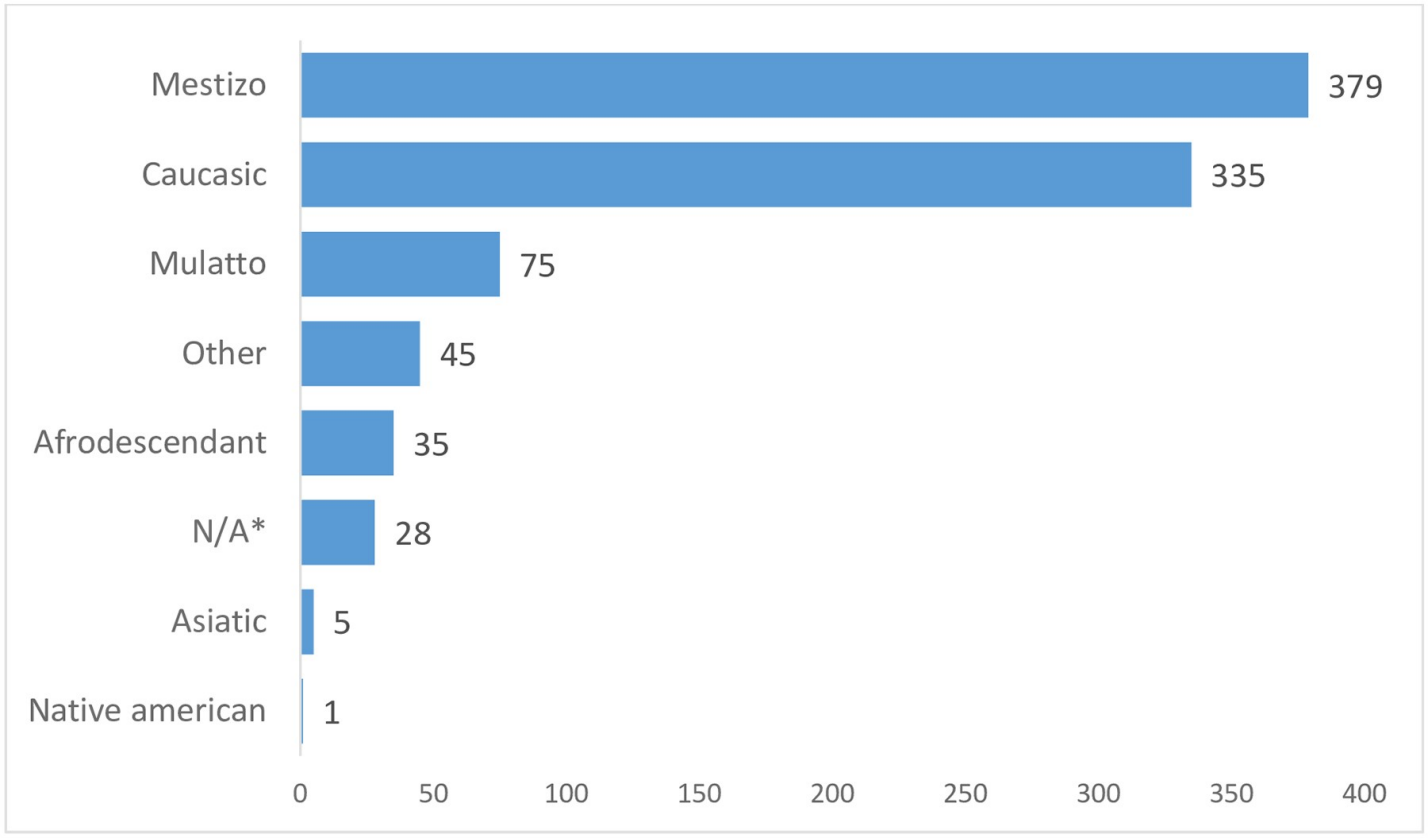
Ethnicity. Mestizo and Caucasian were the predominant ethnic groups. It should be noted that other frequent ethnicities in Latin America were underrepresented.

Nine-hundred and five patients were included in the study. Median age was 64 (50–74) years. Only 21 patients were under 18 years of age. Body mass index (BMI) was 25.8 (22.8–28.6). Five-hundred-fifty patients were male (61%). Distribution of patients by country is depicted in Fig 2. The predominant ethnical groups were Mestizo (379, 42%) and Caucasian (335, 37%) (see Fig 3).

#### Socioeconomic characteristics

The majority of patients (739, 81%) lived in bricked or similar houses, 630 (70%) of which with solid roof. Drinking water was available for 828 patients (91%) and through pipelines for 768 of them (85%). Sewage system was available for 815 individuals (90%). In-house bathroom was present in the houses of 807 patients (90%). The majority of the patients had electricity at home (836, 92%).

#### Characteristics of health coverage, accessibility and first consultation

Most of patients had health coverage provided by the state (556, 61%), 126 (14%) had prepaid system, 122 (13%) had coverage by private health insurance and 47 (5%) had no coverage at all (for 54 patients the information was not available). Accessibility was explored through the way of transportation to the healthcare center. Only 4% did it on foot, bicycle or horse, the remaining patients by public or own motorized vehicle.

#### Acute kidney injury

**Fig 4:**
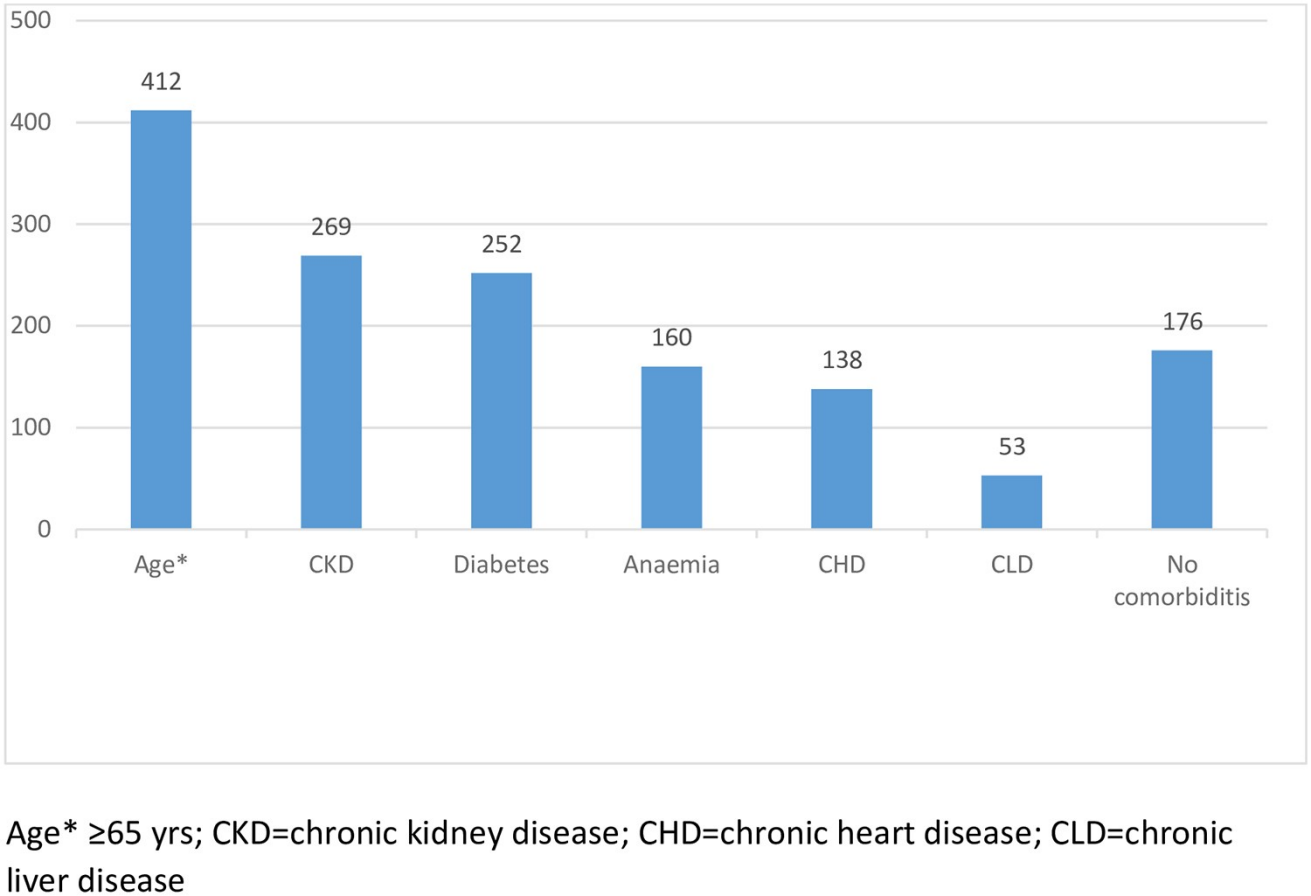
Inherent risk factors for AKI. Comorbidities were very frequent in our population. Only 176 patients that represent 19% of the total were previously healthy.

**Fig 5:**
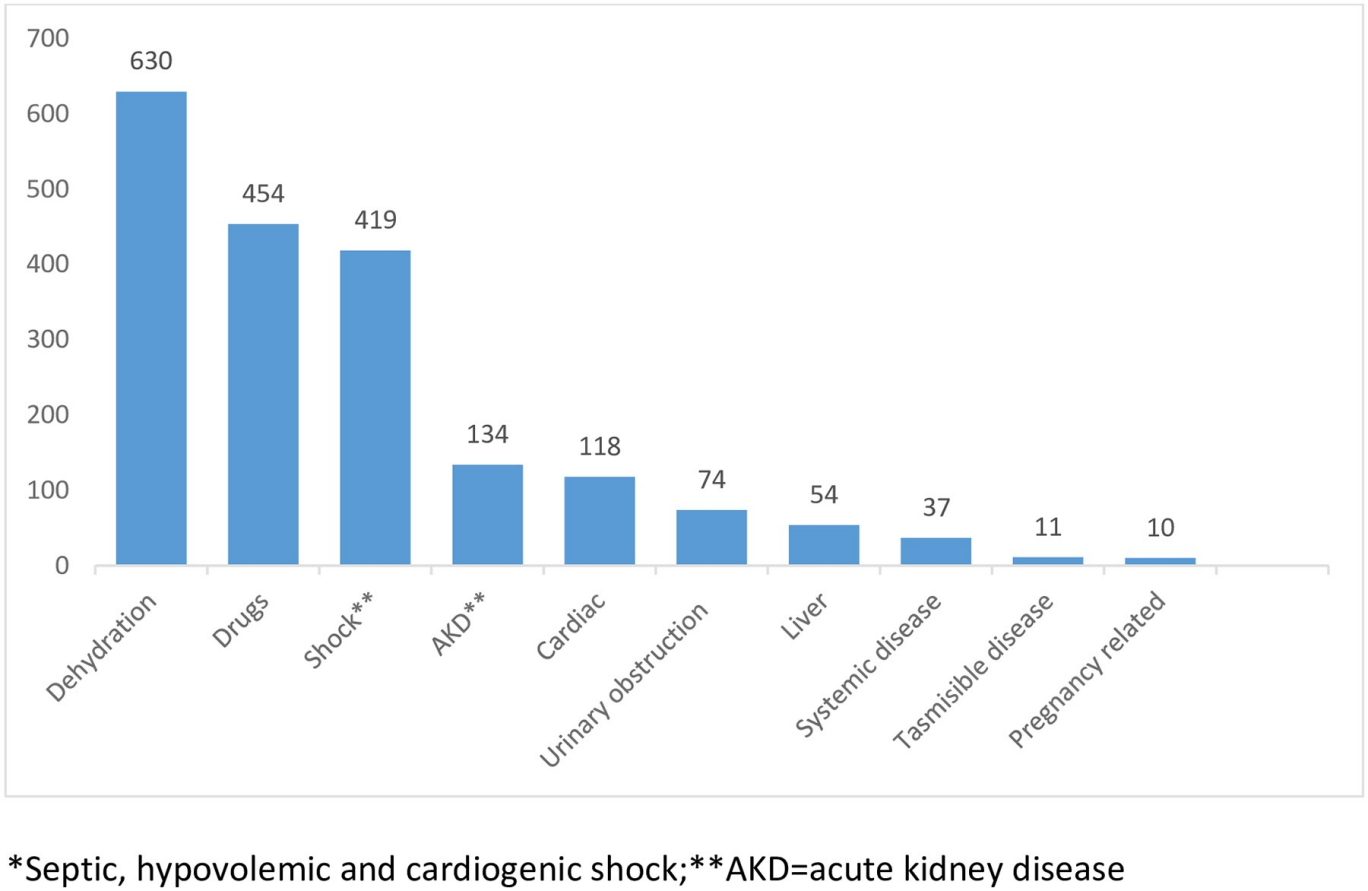
Etiological risk factors for AKI; single or in combination. Dehydration, nephrotoxic drugs and shock were prevalent causes of AKI. The low number of cases related to transmissible diseases and pregnancy complications should be highlighted.**)**, being dehydration (74% vomiting/diarrhea), shock (52% septic) and use of nephrotoxic drugs the most common causes.

Community-acquired AKI (CA-AKI) occurred in 514 cases (62%) and hospital-acquired AKI (HA-AKI) in the remaining 313 patients (38%). In 80 cases, this data was non-available. In patients with CA-AKI, the time between the onset of the disease and the consultation was median (IQR) 4 days (2–8) with 75^th^ percentile greater than 8 days. In 730 patients (81%), one or more inherent risk factors were reported as shown in Fig 4. The etiological risk factors of AKI are shown in Fig 5.

Table 1 depicts the comparison of different variables of CA-AKI *vs*. in HA-AKI. Only variables with significant difference are shown.

**Table 1:** Clinical characteristics of patients by AKI setting.

	CA-AKI (514)	HA-AKI (313)	*P*
Causes of AKI n (%)			
Dehydration	264 (51.4)	106 (33.9)	<0.001
Shock	182 (35.4)	160 (51.1)	<0.001
Cardiac disease	75 (14.6)	63 (20.1)	0.025
AKD	129 (25.1)	25 (8.0)	<0.001
Infection	130 (25.3)	25 (8.0)	0.015
NSAID	75 (14.6)	30 (9.6)	0.022
ACEI	64 (12.5)	30 (9.6)	0.008
Aminoglycosides	13 (2.5)	21 (6.7)	0.003
Vancomycin	16 (3.1)	30 (9.6)	<0.001
Contrast agents	12(2.3)	31 (9.9)	<0.001
Reference SCr, median (IQR)	1.62 (1.00–3.00)	1.20 (0.86–2.00)	<0.001
Pre-dialysis SCr, median (IQR)	5.00 (3.10–7.56)	3.72 (2.21–5.82)	0.001
Kidney replacement therapy	112 (22.6)	103 (36.1)	<0.001
Mechanical ventilation	81 (18.3)	122 (46.9)	<0.001
In-hospital mortality	94 (20.2)	92 (43.8)	<0.001

CA-AKI = community-acquired acute kidney injury; HA-AKI = hospital-acquired acute kidney injury; AKD = Acute Kidney disease (glomerular, vascular, interstitial disease), NSAID = non-steroid antiinflamatory agents; ACEI = angiotensin converting enzyme inhibitors.

The majority of patients were hospitalized in the wards (439, 48%), 254 (28%) in the intensive care unit (ICU) and 108 (12%) in the emergency room. Eighteen patients (2%) were attended in an outpatient health center. No information was provided in 88 cases. Values of reference SCr, SCr at diagnosis, peak SCr, pre-dialysis SCr and SCr at discharge in survivors were median (IQR) 1.50 (0.99–2.80), 2.40 (1.55–3.80), 3.00 (1.81–4.70), 4.30 (2.80–6.90), 1.30 (1.00–2.17) mg//dL, respectively.

The most common hospital complication was infection (437, 52%), with lung (44%), kidney / urinary (24%) and abdomen (16%) as the main frequent sites of infection. Sepsis was observed in 115/885 cases (13%) and organ dysfunctions other than AKI were hemodynamic (90%), respiratory (57%), neurological (35%) and hematological (24%). Fluid overload was reported in 41 patients (4%) and 26 patients (3%) had to undergo surgery due to complications. Nephrology consultation occurred in 633 of 871 patients (73%). Table 2 shows the profile of patients according to consultation with nephrologist.

**Table 2:** Clinical characteristics, process of care, and outcomes of patients by nephrologist consultation during hospitalization.

	Consultation 633	No consultation 238	P
CKD n (%)	231 (36.5)	28 (11.7)	<0.001
HA-AKI n (%)	240 (37.9)	63 (26.4)	0.001
Hypotension/shock n (%)	284 (44.9)	70 (29.4)	<0.001
AKD n (%)	124 (19.6)	33 (13.9)	0.030
Reference SCr mg/dL	2.55±2.56	1.81±1.88	<0.001
SCr at diagnosis	3.54±2.80	2.30±1.82	<0.001
Peak SCr	4.34±3.00	2.28±1.61	<0.001
SCr at discharge	2.14±1.76	1.21±0.72	<0.001
AKI 3	294 (46.4)	39 (16.3)	<0.001
Complications during hospital stay n (%)			
Infection	331 (52.2)	102 (42.8)	0.001
Sepsis	101 (16.0)	14 (5.9)	<0.001
Fluid overload	35 (5.5)	6 (2.5)	0.004
Process of care n (%)			
Fluids iv	519 (82.0)	218 (92.6)	0.008
Diuretics	244 (38.5)	61 (25.6)	<0.001
Vasopressors	197 (31.1)	47 (19.7)	<0.001
Artificial nutrition	214 (33.8)	26 (11.1)	0.003
KRT	180 (28.4	41 (17.2)	<0.001
Mechanical ventilation	519 (82.0)	218 (92.6)	0.007
In-hospital mortality n (%)	165 (26.1)	44 (18.4)	0.004
Renal function at 90-days follow up, n = 228	134	94	
Complete renal recovery n = 119	62 (46.2)	57 (60.6)	0.032

CKD = chronic kidney disease; HA-AKI = hospital-acquired acute kidney injury; AKD = acute kidney disease; KRT = kidney replacement therapy.

Therapy in the general population included intravenous fluids in 88% of patients, antimicrobials in 76%, diuretics in 50%, vasoactive drugs in 34% and enteral or parenteral nutritional support in 36%. Kidney replacement therapy was performed in 242 patients out of 832 (29%) with available data. The most common procedure was intermittent hemodialysis (68%), followed by prolonged intermittent renal replacement therapy (22%), continuous renal replacement therapy (10%), slow continuous ultrafiltration (8%), and peritoneal dialysis (5%). Several patients received more than one procedure. Reasons for initiation of KRT were electrolyte and / or acid base disturbances (70%), fluid overload (54%) and solute control (48%). Fifty-seven patients did not receive KRT despite being indicated; causes for not initiating KRT were death before implementing therapy in 29 (51%), futility in 20 (35%), refused by the patient or relatives due to cultural or religious reasons in 18 (32%) and lack of financial resources in the remaining 5 patients (9%). In 8 patients, 2 or more causes for not initiating KRT were reported. Mechanical ventilation over 24 hours was implemented in 221 patients out of 747 with data (30%).

#### Outcomes

Crude mortality rate of the series was 26% (209 cases). Twenty-three patients were transferred to another hospital, and information of the outcome was not provided in 93 cases. The most frequent causes of death were infection (43%), shock (25%), cardiovascular disease (13%), and malignant disease (4%). Table 3 shows the comparison of sociodemographic and clinical variables between survivors and non-survivors, and Table 4 shows the results of multivariate analysis, with variables independently associated to mortality.

**Table 3:** Risk factors for in-hospital mortality. Univariate analysis.

	Survivors 580	Non-survivors 209	P
Age yrs median (IQR)	62 (50–73)	68 (56–78)	0.001
Inherent risk factors n (%)			
Age≥65	253 (43.6)	120 (57.4)	<0.001
Chronic liver disease	28 (4.8)	21 (10.0)	0.008
Chronic cardiac disease	79 (13.6)	45 (21.5)	0.006
Etiological risk factors n (%)			
Dehydration	275 (47.4)	70 (33.5)	<0.001
Hypotension/shock	188 (32.4)	133 (63.6)	<0.001
Cardiac event	88 (15.2)	54 (25.8)	0.001
Infection	127 (21.9)	72 (34.4)	<0.001
Acute kidney disease	122 (21.0)	21 (10.0)	<0.001
Urinary obstruction	87 (15.0)	15 (7.2)	0.002
Hospital-acquired AKI n (%)	172 (31.7)	92 (49.5)	<0.001
ICU stay n (%)	103 (18.6)	105 (52.2)	<0.001
In-hospital complications n (%)			
Infection	236 (43.2)	149 (74.9)	<0.001
Sepsis	35 (6.0)	69 (33.0)	<0.001
MOD			
Cardiovascular	29 (5.0)	65 (31.1)	<0.001
Pulmonary	16 (2.8)	43 (20.6)	<0.001
Hematological	6 (1.0)	21 (10.0)	<0.001
Neurological	3 (0.5)	35 (16.7)	<0.001
Fluid overload	34 (5.9)	4 (1.9)	<0.001
Nephrologist consultation n (%)	402 (69.8)	165 (79.7)	0.022
Peak SCr mg/dL	2.73 (1.70–4.68)	3.41 (2.37–4.74)	0.001
Pre-dialysis SCr mg/dL	5.10 (3.10–7.70)	3.60 (2.25–5.65)	0.002
AKI stage 3 n (%)	183 (31.6)	117 (56.0)	<0.001
Process of care n (%)			
Diuretics	233 (42.5)	141(70.5)	<0.001
Vasopressors	124 (21.4)	143 (68.4)	<0.001
Antibiotics	386 (70.4)	177 (87.2)	<0.001
Artificial nutrition	110 (20.1)	130 (68.8)	<0.001
KRT	110 (20.1)	106 (53.8)	<0.001
Mechanical ventilation	72 (14.8)	119 (63.3	<0.001

MOD = multiorgan dysfunction; KRT = kidney replacement therapy

**Table 4:** Risk factors independently associated to mortality. Multivariate analysis.

	OR	CI 95%	*P*
Age	1.018	1.006–1.031	0.005
Chronic liver disease	2.510	1.147–5.492	0.021
Etiological causes			
Hypotension/shock	1.923	1.170–3.162	0.010
Cardiac	1.978	1.143–3.423	0.015
Complications			
Sepsis	2.989	1.599–5.586	0.001
Process of care			
KRT	1.983	1.208–3.256	0.007
Mechanical ventilation	3.941	2.334–6.653	<0.001

Data of 90-days follow-up was provided for 413 patients but the information was not complete in all cases. Eighty-eight out of 366 patients with information on vital condition were dead (24%). Renal function was reported in 253 patients: complete recovery occurred in 127 (50%), partial recovery in 77 (30%) and non-recovery in 49 (19%). Eleven patients out of 325 were on dialysis (3.4%). Data on SCr at the end of follow-up was obtained in 235 cases, showing median (IQR) value of 1.20 mg/dL (0.90–1.75). A positive correlation of 90-days SCr was found with the reference, diagnosis, peak and discharge SCr values (S1a, S1b, S1c and S1d Fig. Correlation of 90-days serum creatinine with reference serum creatinine).

## Discussion

There is paucity of data on epidemiology of AKI in Latin America. Some studies were performed in tertiary level hospitals from large cities [6,7,8]. Others have focused on specific conditions such as tropical disease, envenomation by snakebites, spiders, bees, and pregnancy complications [9–12]. The SLANH-AKI Committee carried out a study focused on replacement therapy resources which described by the first time data on equipment and human resources devoted to KRT in the region but providing little information on patients [13]. Recently, Chavez-Iñiguez *et al* [14] published a systematic review including 61 studies from 10 countries (most of them from Brazil) with a total number of 10,670 patients. Although it is a valuable contribution to the knowledge of AKI in the region, representativeness of those results is questionable given the heterogeneity of the studies included. The Global Snapshot study of the ISN 0by25 initiative, planned to assess the diversity of AKI in different settings worldwide [15], showed that only 15% of the 45 centers from Latin America came from low and lower-middle income countries, which together with the low number of cities under 100,000 inhabitants, entailed a sample bias, reflecting the difficulties to recruit patients from small cities and rural areas.

The relationship of AKI with socioeconomic and sanitary conditions is undeniable, however to date there are not accurate and reliable indicators to assess the effect of the aforementioned dimension on AKI profile. Available indicators such as Word Bank GNI classification, Human Development Index and Gini Index, have limitations since they do not reflect the heterogeneity of conditions within each country. For this reason, we collected information on housing, sanitation, drinking water and electricity for each patient, which are four of the six unsatisfied basic needs defined by the Economic Commission for Latin America and the Caribbean, UN, (CEPAL in Spanish) [16]. According to these indicators, less than 13% of patients were in poor housing conditions in contrast with the 21% reported by CEPAL [17]. This difference could be an indicator of limited access to medical care in the poor population but due to study design is a question that we are not able to answer and need further investigation.

Our population was elderly, with high burden of comorbidities and frequently critically ill. These features that mimic AKI from developed world, reflect a high proportion of patients coming from large urban tertiary hospitals, composing the “double epidemiological profile” of AKI in developing world. In this region coexists the typical young and previously healthy patient with AKI related to environmental and socioeconomically conditions, with the old and previously ill patient with AKI mostly acquired in the hospital [18]. In the majority of our cases, AKI was acquired in the community (62%) with late consultation entailing the loss of a window of opportunity for the prevention or mitigation of AKI. Prevalent etiological factors were dehydration, and drugs such as NSAID and ACEI, in contrast with HA-AKI which presented shock, infection, use of antibiotics and intravenous contrast agents as the main causes. Given that prevalent risk factors in CA-AKI are potentially reversible, prevention through education addressed to primary care health care workers and general public is an urgent task. With this aim, the AKI-SLANH Committee carried out an educational strategy addressed to both nephrologists and primary care health workers [19].

Some aspects of the process of care should be mentioned. Patients were seen by nephrologists in 73% of cases. As expected, those with chronic kidney disease, nephropathy as cause of AKI, and HA-AKI were more frequently cared by these specialists. Also, this subset of patients was more prone to receive diuretics, vasopressors, nutritional support, mechanical ventilation and KRT, and had a higher in-hospital and 90-day mortality. According to these results, it is possible to speculate that nephrologist consultation was based rather on the severity of patient’s condition than in a timely and scheduled participation.

Intermittent hemodialysis was the most used KRT, probably due to economical and organizational reasons. Surprisingly, peritoneal dialysis (PD) was underutilized despite its many advantages, including less infrastructure and training required, safety, low cost, better preservation of systemic and renal hemodynamics and lower inflammatory response [20,21]. Our previous report on equipment and human resources in the region [13] described similar results.

Crude in-hospital mortality of our series was high (26%) and not much can be done to improve it since the associated risk factors are little modifiable (age, chronic hepatic disease, shock, sepsis, need of mechanical ventilation and KRT). A mortality rate of 24% at 90-days seems high raising the suspicion of a poor post-discharge care. Nevertheless, this result should be analyzed in light of the great variation reported in prior studies, ranging from 25.6% to 44.6% according the populations under study [22, 23, 24]. Beyond this, the most relevant feature is the high number of patient lost to follow up which denotes the lack of an organized health system ensuring the continuity of the process of care. Finally, regarding renal function at 90-days follow-up, 18.8% of our patients had no recovery, which was associated only to SCr level at the four time-points of the disease course.

Our study has limitations. First, participation was on voluntary basis by open invitation via SLANH which limits the access to the universe of potential patients to by studied. Nevertheless, the number of participants coming from 15 of the 21 countries members of SLANH could be acceptable considering the lower representativeness of previous studies. Second, our results describe the characteristic of a biased sample of cases of AKI in Latin America rather than the epidemiology of AKI in our region; therefore, it cannot be directly extrapolated to other populations. Third, follow-up time was short which limits the information of long-term consequences of AKI. The main strength of our study is that demonstrated the feasibility of studies collecting large amount of information in order to improve the knowledge of the epidemiology of AKI, much of which has not been reported previously in the region and other disadvantaged populations.

In conclusion, our study provides some information that contributes to a better knowledge of AKI in Latin America and the Caribbean. In brief, AKI occurred mainly in the community, in patients with comorbidities and linked to fluid loss and nephrotoxic drugs. Mortality was high, and long-term follow-up poor. Of note, traditional causes of AKI in low-income countries were not prevalent. The paucity of information on the epidemiology of AKI in disadvantaged countries and particularly in Latin America jeopardizes the capacity to struggle against this multidimensional and devastating condition properly characterized as a “human right case” by the ISN 0by25 Initiative. There is an urge to undertake a global strategy involving scientific organizations, international health care agencies and organizations, governments and ministries of health, in order to face this challenge. We hope that our study encourage other colleagues, particularly those involved in the primary care of patients, to pursue in this line of work.

## Supporting information

S1 Fig Correlation of 90-days serum creatinine with reference serum creatinine. (TIF)

## References

[1] Lameire NH, Bagga A, Cruz D, De Maeseneer J, Endre Z, Kellum JA et al Acute kidney injury: an increasing global concern. Lancet. 2013;382(9887):170–179. 10.1016/S0140-6736(13)60647-9 23727171

[2] Lewington AJ, Cerda J, Mehta RL. Raising awareness of acute kidney injury: a global perspective of a silent killer. *Kidney Int* 2013;84: 457–67 10.1038/ki.2013.153 23636171 PMC3758780

[3] Mehta RL, Cerda J, Burdmann EA, Tonelli M, García-García G, Jha V, et al International Society of Nephrology’s 0by25 initiative for acute kidney injury (zero preventable deaths by 2025): a human rights case for nephrology. *Lancet* 2015; 385: 2616–43. 10.1016/S0140-6736(15)60126-X 25777661

[4] Lombardi R, Yu L, Younes-Ibrahim M, Schor N, Burdmann E. A. Epidemiology of acute kidney injury in Latin America. *Semin*. *Nephrol*. 28, 320–329 (2008). 10.1016/j.semnephrol.2008.04.001 18620955

[5] KDIGO AKI Work Group. KDIGO clinical practice guideline for acute kidney injury. Kidney Int Suppl. 2012;2:1–138.

[6] Firmat J, Zucchini A, Martin R, Aguirre C. A study of 500 cases of acute renal failure (1978–1991) Ren Fail. 1994;16:91–9. 10.3109/08860229409044850 8184149

[7] Vukusich A, Alvear F, Villanueva P, González C, Olivari F, Alvarado N, et al Epidemiology of severe acute renal failure in Metropolitan Santiago Epidemiología de la Insuficiencia Renal Aguda grave. Un studio prospectivo multicéntrico en la Región Metropolitana. Rev Med Chile 2004; 132: 1355–1361 *(Spanish)* 15693197

[8] Palomba H, do Amaral Campos PP, Corrêa TD, de Carvalho FB, Westphal G, Gusmão D, et al Defining and treating acute kidney injury patients in Brazilian intensive care units: Results from a cross-sectional nationwide survey. J Crit Care. 2016 8;34:33–7. 10.1016/j.jcrc.2016.03.018 27288607

[9] Daher EF, Silva GB Jr, Lima RSA, Mota RMS, Rocha HAL, de Abreu KLS, et al Different Patterns in a Cohort of Patients with Severe Leptospirosis (Weil Syndrome): Effects of an Educational Program in an Endemic Area. Am J Trop. Med Hyg, 2011 85:479–484 10.4269/ajtmh.2011.11-0080 21896808 PMC3163870

[10] Pinho FMO, Zanetta DMT, Burdmann EA. Acute renal failure after *Crotalus durissus* snakebite: A prospective survey on 100 patients. Kidney Int 2005, 67:659–667 10.1111/j.1523-1755.2005.67122.x 15673314

[11] Lucato RV Jr, Abdulkader RC, Barbaro KC, Mendes GE, Castro I, Baptista MA, et al Loxosceles gaucho venom-induced acute kidney injury—in vivo and in vitro studies. PLoS Negl Trop Dis. 2011;5:e1182 10.1371/journal.pntd.0001182 21655312 PMC3104973

[12] Bezerra da Silva GB Jr, Saintrain SV, Castelo GC, de Vasconcelos VR, de Oliveira JGR, Rocha AMT, et al Acute kidney injury in critically ill obstetric patients: a cross-sectional study in an intensive care unit in Northeast Brazil. Braz. J. Nephrol 2017;39:357–361. 10.5935/0101-2800.20170066 29319760

[13] Lombardi R, Rosa-Diez G, Ferreiro A, Greloni G, Yu L, Younes-Ibrahim M, et al; on behalf of the Acute Kidney Injury Committee of the Latin American Society of Nephrology and Hypertension (SLANH)Working Group. Acute kidney injury in Latin America: a view on renal replacement therapy resources. Nephrol Dial Transplant (2014) 29: 1369–1376 10.1093/ndt/gfu078 24744281

[14] Chavez-Iñiguez JS, García-García G, Lombardi R. Epidemiología y desenlaces de la lesión renal aguda en Latinoamérica. Gac Med Mex. 2018;154(Supp 1):S6–S14. Spanish. 10.24875/GMM.M18000067 30074021

[15] Mehta RL, Burdmann EA, Cerdá J, Feehally J, Finkelstein F, García-García G, et al Recognition and management of acute kidney injury in the International Society of Nephrology 0by25 Global Snapshot: a multinational cross-sectional study. Lancet. 2016 14;387:2017–25. 10.1016/S0140-6736(16)30240-9 27086173

[16] Fresneda O. La medida de necesidades básicas insatisfechas (NBI) como instrumento de medición de la pobreza y focalización de programas. Serie Estudios y Perspectivas No 18, Bogotá, 2007. CEPAL, Naciones Unidas.

[17] Szalachman R. Perfil de déficit y políticas de vivienda de interés social: Situación de algunos países de la región en los noventa. Serie Financiamiento del desarrollo. Santiago de Chile, 2000, CEPAL, Naciones Unidas

[18] Kashani K, Macedo E, Burdmann EA, Hooi LS, Khullar D, Bagga A, et al; on behalf of the Acute Disease Quality Initiative (ADQI) Consensus Group. Acute Kidney Injury Risk Assessment: Differences and Similarities Between Resource-Limited and Resource-Rich Countries. Kidney Int Rep (2017) 2, 519–529 10.1016/j.ekir.2017.03.014 28845471 PMC5568820

[19] Lombardi R, Ferreiro A, Rosa-Diez G, Margolis A, Yu L, Younes-Ibrahim M, et al Raising Awareness of Acute Kidney Injury: A Latin American Experience. Kidney Int Rep (2018) 3, 1416–1423 10.1016/j.ekir.2018.08.003 30450468 PMC6224626

[20] Gabriel DP, Caramori JT, Martim LC, Barretti P Balbi AL. High volume peritoneal dialysis vs daily hemodialysis: A randomized, controlled trial in patients with acute kidney injury. Kidney Intern 2008,73: S87–S93 10.1038/sj.ki.5002608 18379555

[21] Ponce D, Buffarah MB, Goes C, Balbi A. Peritoneal Dialysis in Acute Kidney Injury: Trends in the Outcome across Time Periods. PLoS One. 2015 12;10:e0126436 10.1371/journal.pone.0126436 25965868 PMC4428622

[22] Nisula S, Kaukonen KM, Vaara ST, Korhonen AM, Poukkanen M, Karlsson S, et al; FINNAKI Study Group. Incidence, risk factors and 90-day mortality of patients with acute kidney injury in Finnish intensive care units: the FINNAKI study. Intensive Care Med. 2013,39:420–428 10.1007/s00134-012-2796-5 23291734

[23] RENAL Replacement Therapy Study Investigators, Bellomo R, Cass A, Cole L, Finfer S, Gallagher M, Lo S, et al Intensity of continuous renal-replacement therapy in critically ill patients. N Engl J Med 2009, 61:1627–1638 10.1056/NEJMoa0902413 19846848

[24] Holmes J, Rainer T, Geen J, Roberts G, May K, Wilson N, et al; on behalf of the Welsh AKI Steering Group. Acute Kidney Injury in the Era of the AKI E-Alert. Clin J Am Soc Nephrol 11: 2123–2131, 2016 10.2215/CJN.05170516 27793961 PMC5142071

