# Supplementary figures and images for: EPILAT-IRA Study: A contribution to the understanding of the epidemiology of acute kidney injury in Latin America
Source: PLoS One. 2019 Nov 14;14(11):e0224655. doi: 10.1371/journal.pone.0224655 (PMC6855418; doi:10.1371/journal.pone.0224655)

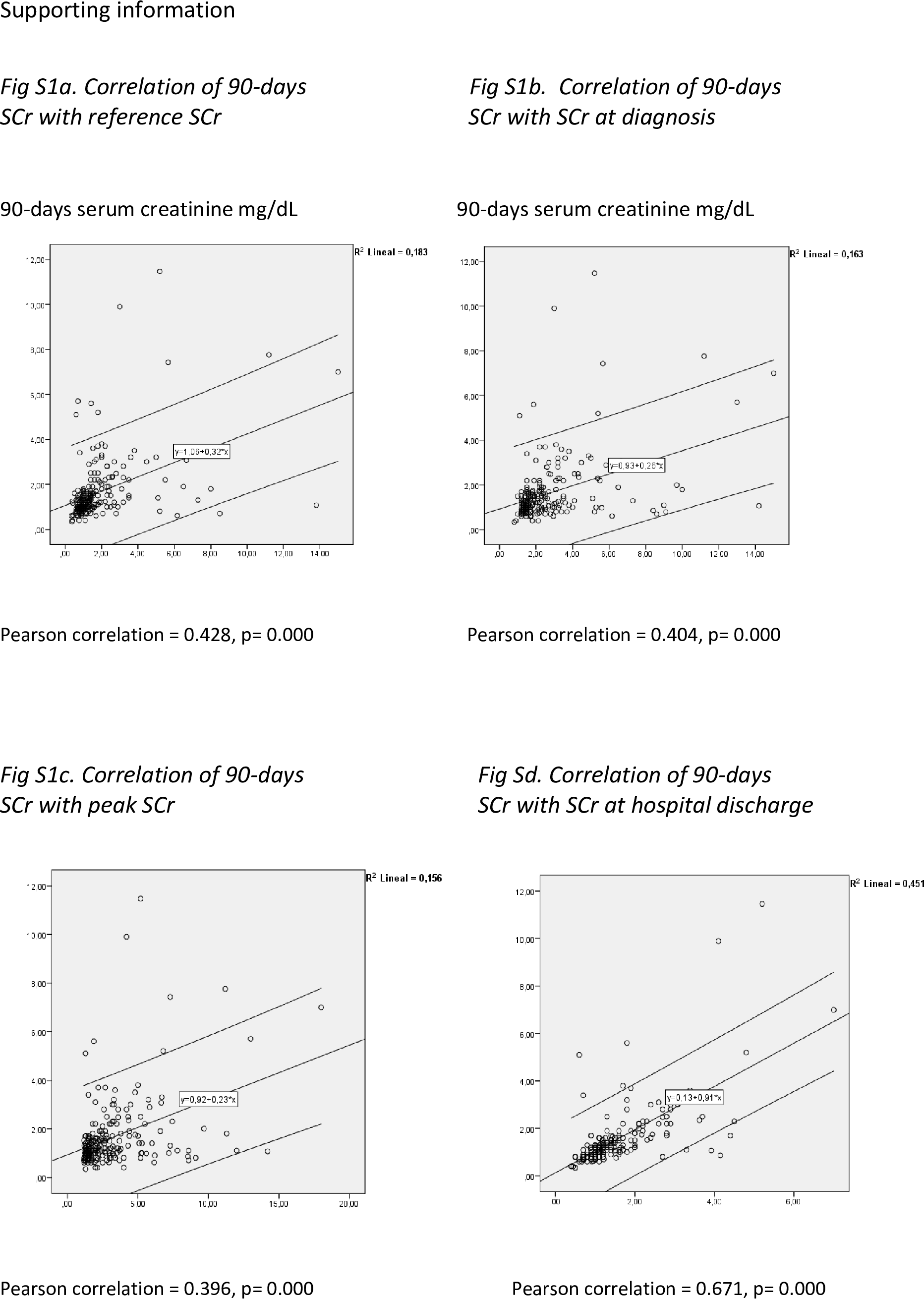

Supplement: S1 Fig — (TIF) [file pone.0224655.s001.tif]
